# The Impact of the hsCRP/BMI Ratio on Cardiovascular Outcomes in CAD Patients: A Population-Based Study

**DOI:** 10.1155/mi/6082331

**Published:** 2025-11-24

**Authors:** Da Liu, Xuliang Wang, Kuo Zhang, Xiangbin Meng, Jingjia Wang, Jie Yang, Jun Gao, Yuangengshuo Wang, Jun Wen, Chen Li, Mingqi Zheng, Gang Liu, Yue Ma, Wenyao Wang, Yi-Da Tang, Chunli Shao

**Affiliations:** ^1^The Second Affiliated Hospital, Southern University of Science and Technology, Shenzhen 518055, China; ^2^State Key Laboratory of Vascular Homeostasis and Remodelling, NHC Key Laboratory of Cardiovascular Molecular Biology and Regulatory Peptides, Beijing Key Laboratory of Cardiovascular Receptors Research, Department of Cardiology, Institute of Vascular Medicine, Peking University Third Hospital, Peking University, Beijing 100191, China; ^3^State Key Laboratory of Cardiovascular Disease, Department of Cardiology, Fuwai Hospital, National Center for Cardiovascular Diseases, Chinese Academy of Medical Sciences and Peking Union Medical College, Beijing 100037, China; ^4^Department of Cardiology, The First Hospital of Hebei Medical University, No. 89 Donggang Road, Shijiazhuang, Hebei 050000, China

**Keywords:** BMI, CAD, DES, high-sensitivity C-reactive protein, inflammation

## Abstract

**Background:**

It remains uncertain whether a novel composite indicator, the high sensitivity C-reactive protein (CRP) (hsCRP)-to-body mass index (BMI) ratio (CBR), is associated with unfavourable events in subjects who have undergone drug-eluting stent (DES) implantation. Therefore, we conducted a prospective cohort study to evaluate the associations between the CBR and adverse outcome occurrences.

**Methods:**

From January 2013 to December 2013, a total of 9810 subjects who underwent DES implantation were enrolled in the study. The participants were divided into three groups on the basis of the CBR. We defined the primary endpoint as the occurrence of major adverse cardiovascular and cerebrovascular events (MACCEs), and the secondary endpoints included the occurrence of all-cause death, myocardial infarction(MI), stroke or target vessel revascularization (TVR). Kaplan‒Meier (K‒M) analysis and Cox regression analysis were performed to assess the discrepancy in the risk of adverse outcomes between the different CBR groups.

**Results:**

Over an average follow-up period of 26.7 months, 1022 MACCEs were recorded, which included 122 deaths, 392 MIs, 156 strokes and 461 instances of TVR. The results from the K‒M analysis suggested that the rates of MACCEs and MI increased with increasing tertiles of the CBR. Furthermore, Cox regression analysis demonstrated that there were significant associations between a high CBR and increased MACCE and MI risk.

**Conclusion:**

In subjects who underwent DES implantation, a higher CBR was significantly related to increased long-term MACCE and MI risk.

## 1. Introduction

Cardiovascular disease (CVD) remains the predominant cause of mortality globally, accounting for a substantial number of deaths each year [1, 2]. Although secondary prevention therapy, including lifestyle intervention, medicine, vascular intervention and surgery, has been significantly improved and popularised, the mortality rate of coronary artery disease (CAD) remains high [3]. In recent years, cholesterol levels in patients with CAD have significantly decreased due to the widespread use of protein convertase subtilisin/kexin type 9 (PCSK9) inhibitors [4], whereas the risk of residual inflammation has slowly increased. C-reactive protein (CRP) is a common biomarker of systematic inflammation that can induce subintimal cholesterol deposition and facilitate atherosclerosis development [5–7]. High-sensitivity CRP (hsCRP) is particularly sensitive for detecting low-grade inflammation in humans. Numerous investigations have shown that CRP is intimately linked to cardiovascular outcomes in patients with CAD. Elevated levels of CRP have been linked to an increased risk of adverse cardiovascular outcomes, including myocardial infarction (MI), stroke and heart failure [8, 9]. Additionally, numerous studies have indicated a correlation between body mass index (BMI) and the levels of inflammation as well as cardiovascular events in patients with CAD [10–12]. Recently, investigations have been conducted to examine the impact of hsCRP levels on cardiovascular outcomes, categorised according to BMI, in individuals diagnosed with CAD [13, 14]. Jia et al. [15] proposed a composite marker, the hsCRP-to-BMI ratio (CBR), which could adjust for the effect of BMI on hsCRP levels and was reported to be related to cardiovascular event risk in subjects with acute coronary syndrome (ACS). However, it remains unclear whether the CBR is independently associated with long-term cardiovascular event risk in CAD patients who have undergone drug-eluting stent (DES) implantation. Consequently, we conducted a population-based study to assess the predictive value of the CBR for cardiovascular prognosis in patients undergoing DES implantation.

## 2. Methods

### 2.1. Study Protocol and Population

The present study was designed as a prospective, observational cohort study in strict compliance with the ethical principles outlined in the Declaration of Helsinki. Moreover, the study protocol was sanctioned, and the research process was overseen by the ethics review committee of Fuwai Hospital, Beijing, China (Number 2012-431). Written informed consent was obtained from all participants.

From January 2013 to December 2013, the researchers continuously screened 10,112 CAD patients who underwent DES implantation at Fuwai Hospital, Beijing, China. Patients who met any of the following criteria were excluded: (1) were younger than 18 years of age, (2) had extreme obesity (BMI >45 kg/m^2^) or cachexia (BMI <15 kg/m^2^), (3) lacked hsCRP or BMI data, (4) had concomitant acute infectious illnesses, autoimmune illness or malignant tumours, (5) had severe renal insufficiency and (6) did not have complete 2-year follow-up outcomes. Details can be found in Supporting Information 1: Figure S1. During our research, a total of 9810 patients with CAD who had undergone DES implantation were included. To further elucidate the associations between the CBR and the occurrence of cardiovascular outcomes, we classified all participants into three distinct groups on the basis of the CBR: tertile 1, which included patients with a CBR of 0.04 or lower (*n* = 3270); tertile 2, comprising patients with a CBR ranging from greater than 0.04 to 0.11 (*n* = 3270) and tertile 3, which consisted of patients with a CBR greater than 0.11 (*n* = 3270). This categorisation enabled us to investigate the impacts of varying CBR levels on cardiovascular risk and outcomes within the patient cohort.

### 2.2. Data Measurement

After individuals signed an informed consent form, investigators meticulously gathered and recorded their baseline data: sex, age, height, weight, principal diagnosis, family history of premature CAD, smoking status, previous history of hypertension (HTN), hyperlipidaemia (HL), diabetes, MI, stroke or transient ischaemic attack, percutaneous coronary intervention (PCI) and coronary artery bypass graft (CABG). In addition, the results of the echocardiogram and laboratory exams were also recorded. Elevated hsCRP was defined as >3 mg/L. Additionally, essential medications at the time of discharge, such as statins, dual antiplatelet therapy (DAPT) and β-blockers, were also documented.

Two independent intervention cardiologists analysed the outcomes of coronary angiography and the intervention and documented the number of coronary arteries exhibiting a constriction of 50% or more, which is unique to the spectrum of coronary artery abnormalities, the SYNTAX score [16] and the characteristics of the implanted stents. BMI was determined via the following equation:

  $$\begin{array}{c} \left[\text{BMI}=\text{weight divided by the square of height }\left({\text{kg/m}}^{2}\right)\right]. \end{array}$$

We defined stroke as ischaemic stroke, cerebral haemorrhage or TIA. We determined the principal diagnosis according to relevant guidelines [17–19].

### 2.3. Follow-Up

The dedicated staff followed-up with all the subjects at 1, 6, 12 and 24 months after PCI by means of outpatient visits, telephone calls and letters. The principal result was characterised as major adverse cardiovascular and cerebrovascular events (MACCEs), an amalgamation that includes death from all causes, nonfatal MIs, nonfatal strokes and target vessel revascularization (TVR). In addition, the secondary endpoints included all-cause death, MI, stroke and TVR. We characterised all-cause mortality as death resulting from any cause. The determination of MIs and strokes adhered to the definitions outlined above. Furthermore, TVR was defined as the act of performing revascularization procedures again in the targeted coronary artery. All participants were monitored for a duration of 2 years post-PCI, except instances where data were missing or in cases of death.

### 2.4. Statistical Analysis

All the statistical analyses and diagram manufacturing were implemented in R software (version 4.1.1) and STATA MP 14.0. We represented continuous variables with either the mean plus or minus standard deviation (SD) or the median (interquartile range), contingent on the outcomes of the normality assessment. Categorical data are represented by numbers and percentages. The chi-square (χ^2^) test was conducted to assess disparities in categorical variables across the three groups. Furthermore, Kaplan–Meier (K‒M) survival analysis coupled with the log-rank test was used to assess the discrepant risks of the primary endpoint and secondary endpoints across the groups categorised by the CBR. In addition, we constructed Cox proportional hazards models to calculate the hazard ratios (HRs) and 95% confidence intervals (CIs) of the CBR both as a continuous variable and as a categorical variable. Furthermore, within the context of multivariable Cox regression analysis, we took steps to account for potential confounding variables that might affect the study results according to clinical experience and outcomes in univariate Cox regression. Additionally, the restricted cubic spline (RCS) method was utilised to evaluate the latent nonlinear relationships between the CBR and the occurrence of cardiovascular outcomes. In the present study, a two-tailed *p*-value less than 0.05 was considered to indicate statistical significance.

## 3. Results

### 3.1. Baseline Data Analysis

Finally, 9810 CAD patients were included in the study. The average age was 58.32 years, 7560 (77.1%) subjects were men, 23.6% of the subjects were obese, 1819 (18.5%) subjects had acute MI (AMI), 6306 (64.3%) patients had previous HTN, 5597 (57.1%) patients were current smokers, 2928 (29.8%) patients had concomitant diabetes mellitus and 2690 (27.4%) participants had previously undergone coronary revascularization. We divided all the participants into three groups on the basis of their CBR tertiles. The specific baseline data of the three groups are presented in Table 1. There were significant baseline discrepancies across the three groups, including sex; age; principal diagnosis; history of HTN, stroke, MI and PCI; severity and complexity of coronary diseases; laboratory testing results (white blood cell count, haemoglobin levels, platelet count, the estimated glomerular filtration rate (eGFR), uric acid levels, and glucose and lipid profiles); and medication at discharge (ACEIs/ARBs, CCBs and DAPT). In addition, we found that a higher CBR was closely associated with older age, male sex, greater AMI risk, a lower left ventricular ejection fraction (LVEF) and eGFR, more complex coronary lesions and worse control of glucose and lipids.

### 3.2. Adverse Cardiovascular Outcomes

**Figure 1:**
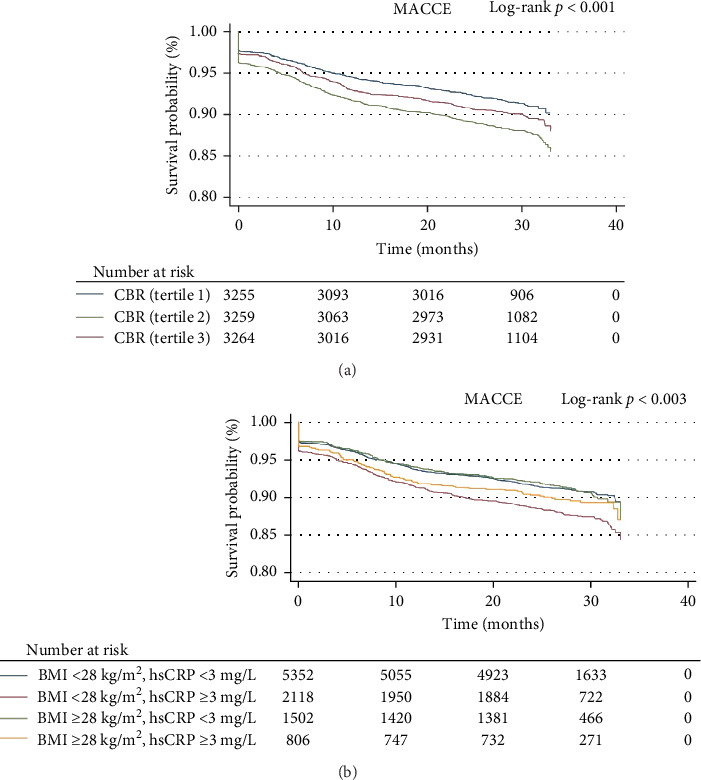
Kaplan‒Meier survival analysis of the primary endpoint in the study population. (a) Population stratified according to the CBR tertilesand (b) population stratified according to elevated hsCRP and BMI. Abbreviations: CBR, hsCRP-to-BMI ratio; MACCEs, major adverse cardiovascular and cerebrovascular events.

**Figure 2:**
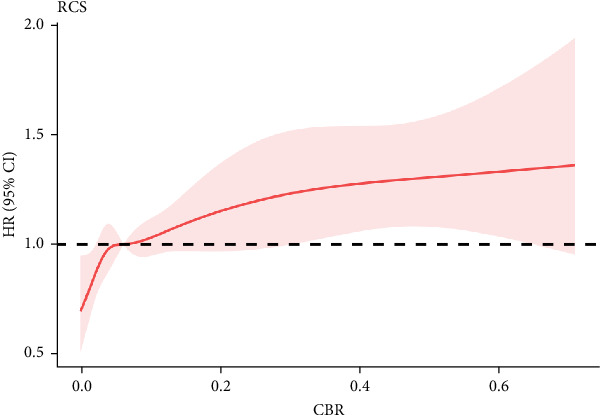
Restricted cubic spline of the association between the CBR and MACCE risk. The multivariable analysis model was adjusted for age, sex, AMI, family history, previous MI, previous CABG, previous PCI, history of HTN, HL, DM, stroke, smoking status, LM disease, 3-vessel disease, CTO disease, SYNTAX score, profiles of stent implantation, IABP application, LVEF, eGFR, WBC, PLT, Hb, HbA1c, FBG, TG, LDL-C, HDL-C, TC, uric acid and medication after discharge. Hazard ratios are indicated by solid lines, and 95% CIs are indicated by shaded areas.

Over an average follow-up period of 26.7 months, investigators documented 1022 MACCEs, 122 deaths from all causes, 392 nonfatal MIs, 156 strokes and 461 TVRs. The findings from the K–M analysis indicated that the incidence rates of MACCEs and MI increased with increasing tertiles of the CBR. Moreover, our study revealed that participants in the group with elevated hsCRP levels but decreased BMI had the lowest MACCE-free survival following risk stratification on the basis of hsCRP and BMI (log-rank *p* = 0.003). The detailed results of the K‒M analysis are shown in Figure 1 and Supporting Information 1: Figure S2. The results of multivariable Cox regression analysis suggested that the CBR was an independent risk factor for MACCEs (tertile 3 vs. tertile 1, HR: 1.27, 95% CI, 1.07–1.50, *p* = 0.005; HR per 1-SD increase: 1.08, 95% CI, 1.01–1.14, *p* = 0.019) and MI (tertile 3 vs. tertile 1, HR: 1.32, 95% CI, 1.03–1.71, *p* = 0.027; HR per 1-SD increase: 1.10, 95% CI, 1.01–1.20, *p* = 0.039) (Tables 2 and 3 and Supporting Information 2: Tables S1 and S2). Other independent risk factors for MACCEs included age (HR: 1.01, 95% CI, 1.002–1.02, *p* = 0.002), previous PCI (HR: 1.25, 95% CI, 1.07–1.46, *p* = 0.004), stroke (HR: 1.30, 95% CI, 1.08–1.56, *p* = 0.006), three-vessel disease (HR: 1.28, 95% CI, 1.11–1.47, *p* = 0.001), SYNTAX score (HR: 1.28, 95% CI, 1.11–1.47, *p* = 0.008), IABP application (HR: 1.96, 95% CI, 1.18–2.91, *p* = 0.007), LVEF (HR, 0.98, 95% CI, 0.97–0.99, *p*  < 0.001), DAPT (HR, 0.37, 95% CI, 0.22–0.63, *p*  < 0.001), and statin use (HR, 0.69, 95% CI, 0.51–0.94, *p* = 0.018). The results of the RCS analysis suggested that the relationships between the CBR and both MACCE and MI risk exhibited roughly linear trends (MACCE: *p* for overall <0.001, *p* for nonlinearity = 0.14; MI: *p* for overall = 0.042, *p* for nonlinearity = 0.28). The detailed RCS outcomes are illustrated in Figure 2 and Supporting Information 1: Figure S3.

## 4. Discussion

In the present study, we delved into the value of the CBR for predicting cardiovascular outcomes in CAD patients who were subjected to DES implantation and arrived at the following conclusions: (1) patients with elevated hsCRP levels but a lower BMI had the lowest MACCE-free survival rate; (2) the CBR was closely related to cardiovascular prognosis, including MACCE and MI risk and (3) the CBR could be used to avoid the confounding influence of BMI on the relationship between hsCRP levels and the occurrence of cardiovascular outcomes.

Inflammation is a significant contributor to the onset and progression of arteriosclerosis, which involves various inflammatory cells, including T lymphocytes, B lymphocytes, monocyte-derived macrophages and mast cells. Lipoproteins with oxidative modifications can activate arterial inflammation, which triggers the release of proinflammatory cytokines, thereby facilitating the recruitment of corresponding inflammatory cells, especially circulating monocytes. An imbalance between proinflammatory and proresolving processes can lead to advanced atherosclerosis [7, 20, 21]. In addition, inflammation is closely related to vulnerable plaques and the severity of coronary stenosis [22, 23] and induces thrombogenesis during AMI [24, 25]. Furthermore, the implantation of a stent can lead to an augmented localised inflammatory response within the artery and impede endothelial healing [26]. Many studies have reported that CRP or hsCRP is a robust risk factor for cardiovascular outcomes in individuals with known CAD [8, 27, 28]. Obesity status is linked to sustained, low-grade inflammation and can potentially trigger the development of metabolic disorders and accelerate ageing [29–31]. Obesity can promote the recruitment of proinflammatory macrophages, which secrete large amounts of TNF-α [32]. Previous investigations have indicated that obesity status, particularly that indicated by a high body fat percentage, is correlated with a less favourable prognosis of CAD [33, 34]. Recently, certain researchers have explored the prognostic significance of hsCRP levels across various BMI categories in CAD patients. Beyhoff et al. [13] analysed data from 14,140 patients who underwent PCI from 2009 to 2017 in the U.S. and classified them into four groups on the basis of their BMI. Patients with AMI, malignant tumours, severe inflammatory conditions or who were underweight were excluded from the final analysis. They defined elevated hsCRP concentrations as higher than 3 mg/L. The primary endpoint was defined as major adverse cardiovascular events (MACEs) at the 1-year follow-up. The incidence of elevated hsCRP levels gradually increased with increasing BMI, and elevated hsCRP levels were consistently related to cardiovascular outcomes across all BMI categories [13]. Ding et al. [14] consecutively recruited 1871 patients with CAD from 2008 to 2011 from three hospitals in China. They divided the participants according to BMI and hsCRP levels, and the median follow-up time was 3.1 years. They characterised the study endpoints as encompassing all-cause mortality and cardiovascular mortality. The results suggested that patients with a normal weight (BMI < 24 kg/m^2^) with elevated hsCRP levels had a 2.54-fold increased risk of all-cause death and a 2.36-fold increased risk of cardiovascular death in comparison to overweight subjects with normal hsCRP levels [14]. Jia et al. [15] investigated the predictive significance of the CBR on cardiovascular prognosis in 478 subjects suffering from ACS throughout an average monitoring duration of 50 months. The primary outcome was defined as MACEs, a composite of cardiac mortality, MI, repeat revascularization, stroke and heart failure. The results indicated that the CBR was independently and positively correlated with MACEs in ACS patients, and individuals with higher hsCRP levels but lower weights presented the lowest MACE-free survival rate, which aligns with our findings. Compared with prior research, the current study concentrated on patients who were subjected to PCI and featured a substantially larger sample along with a more persuasive design of endpoints. Furthermore, RCS was carried out to assess the trend of the connexion between the CBR and the occurrence of cardiovascular outcomes, revealing roughly linear associations. On the whole, numerous scholars have acknowledged the fundamental impact of BMI when they evaluated the predictive value of inflammatory indicators in patients with CAD and endeavoured to devise studies according to varying BMI levels. The novel composite index, the CBR, might be effective in eliminating the confounding effect of obesity on the relationship between hsCRP and cardiovascular prognosis in subjects with CAD.

## 5. Limitations

This study has several limitations. First, certain alternative inflammatory markers, including the neutrophil/lymphocyte ratio and IL-6 level, were not documented or analysed. In addition, lean body mass, waist circumference and the waist-to-hip ratio, which are beneficial for defining and classifying obesity, were not measured in the present study. Moreover, there could be unmeasured confounding elements mediating the relationship investigated in this study, a limitation that is inherent to the observational character of the research design of the present study.

## 6. Conclusion

An increased CBR was an independent risk factor for long-term MACCE and MI risk in subjects who underwent DES implantation. This novel composite index might be effective in eliminating the confounding effect of obesity on the relationship between inflammation and cardiovascular prognosis in subjects with CAD. Future research should include multicentre studies to further explore the predictive value of the CBR in different ethnic groups to gain a broader understanding of its applicability and effectiveness across various populations.

## Figures and Tables

**Table 1:** Baseline demographic and clinical data of three groups.

Variables	Tertile 1 (CBR ≤ 0.04) *N* = 3270	Tertile 2 (0.04 < CBR ≤ 0.11) *N* = 3270	Tertile 3 (0.11 < CBR) *N* = 3270	*p*-Value
CBR	(0.014, 0.031)^a^	(0.050, 0.079)^a^	(0.138, 0.422)^a^	<0.001
Age (years)	57.88 ± 9.86	58.28 ± 10.19	58.75 ± 10.49	0.001
Male, *n* (%)	2597 (79.4%)	2466 (75.4%)	2497 (76.4%)	<0.001
BMI (kg m^2^)	25.73 ± 3.06	26.04 ± 3.15	25.96 ± 3.22	<0.001
Principal diagnosis				
CCS, *n* (%)	1389 (42.5%)	1242 (38.0%)	928 (28.4%)	
UA, *n* (%)	1606 (49.1%)	1543 (47.2%)	1283 (39.2%)	<0.001
NSTEMI, *n* (%)	208 (6.4%)	375 (11.5%)	803 (24.6%)	
STEMI, *n* (%)	67 (2.0%)	110 (3.4%)	256 (7.8%)	
Family history, *n* (%)	760 (23.2%)	805 (24.6%)	799 (24.4%)	0.369
Previous MI, *n* (%)	672 (20.6%)	611 (18.7%)	511 (15.6%)	<0.001
Previous PCI, *n* (%)	877 (26.8%)	756 (23.1%)	672 (20.6%)	<0.001
Previous CABG, *n* (%)	143 (4.4%)	112 (3.4%)	130 (4.0%)	0.140
HTN, *n* (%)	1972 (60.3%)	2169 (66.3%)	2165 (66.2%)	<0.001
Hyperlipemia, *n* (%)	2190 (67.0%)	2214 (67.7%)	2189 (66.9%)	0.757
DM, *n* (%)	951 (29.1%)	963 (29.4%)	1014 (31.0%)	0.195
Previous stroke, *n* (%)	312 (9.5%)	337 (10.3%)	383 (11.7%)	0.015
Current smoker, *n* (%)	1753 (53.6%)	1886 (57.7%)	1958 (59.9%)	<0.001
LM disease, *n* (%)	188 (5.7%)	224 (6.9%)	206 (6.3%)	0.187
3-Vessel disease, *n* (%)	1326 (40.6%)	1397 (42.7%)	1471 (45.0%)	0.001
CTO disease, *n* (%)	229 (7.0%)	210 (6.4%)	209 (6.4%)	0.533
ISR disease, *n* (%)	135 (4.1%)	130 (4.0%)	93 (2.8%)	0.010
SYNTAX score	9.5 (6, 16)^a^	10 (6, 16)^a^	11 (6, 17.5)^a^	<0.001
Number of stents	2 (1, 2)^a^	2 (1,2)^a^	2 (1, 2)^a^	<0.001
Diameter of stents (mm)	3.03 ± 0.53	3.00 ± 0.51	3.01 ± 0.52	0.077
Length of stents (mm)	28 (18, 40)^a^	28 (18, 40)^a^	28 (18, 43)^a^	0.034
IABP, *n* (%)	12 (0.4%)	22 (0.7%)	50 (1.5%)	<0.001
WBC (×10^9^)	7.26 ± 1.91	7.62 ± 1.95	7.96 ± 2.00	<0.001
Hb (g/L)	137.89 ± 14.82	137.31 ± 14.97	134.93 ± 15.26	<0.001
PLT (×10^9^)	186.50 ± 47.57	197.07 ± 51.07	212.17 ± 59.87	<0.001
LVEF (%)	63.81 ± 6.72	63.28 ± 7.17	61.82 ± 7.60	<0.001
eGFR (mL/min/1.73 m^2^)	97.87 ± 18.66	96.14 ± 19.63	94.30 ± 21.13	<0.001
TG (mmol/L)	1.41 (1.04, 1.93)^a^	1.61 (1.20, 2.22)^a^	1.57 (1.19, 2.15)^a^	<0.001
TC (mmol/L)	3.98 ± 1.03	4.26 ± 1.06	4.36 ± 1.09	<0.001
HDL-C (mmol/L)	1.08 ± 0.29	1.03 ± 0.26	0.99 ± 0.26	<0.001
LDL-C (mmol/L)	2.32 ± 0.87	2.54 ± 0.90	2.65 ± 0.92	<0.001
FBG (mmol/L)	5.91 ± 1.78	6.19 ± 2.05	6.60 ± 2.52	<0.001
HbA1c (%)	6.45 ± 1.10	6.62 ± 1.19	6.77 ± 1.36	<0.001
Uric acid (umol/L)	334.16 ± 78.63	346.89 ± 84.31	345.06 ± 89.68	<0.001
HsCRP (mg/L)	0.58 (0.36, 0.80)^a^	1.61 (1.30, 2.05)^a^	5.75(3.62, 11.35)^a^	<0.001
DAPT, *n* (%)	3258 (99.6%)	3257 (99.6%)	3240 (99.1%)	0.004
Statins, *n* (%)	3173 (97.0%)	3170 (96.9%)	3148 (96.3%)	0.163
β-blockers, *n* (%)	2821 (86.3%)	2859 (87.4%)	2891 (88.4%)	0.033
ACEIs/ARBs, *n* (%)	1719 (52.6%)	1878 (57.4%)	1975 (60.4%)	<0.001
CCBs, *n* (%)	1595 (48.8%)	1606 (49.1%)	1441 (44.1%)	<0.001

*Note*: CBR, hsCRP-to-BMI ratio; SYNTAX, synergy between PCI with TAXUS and cardiac surgery.

Abbreviations: ACEIs, angiotensin-converting enzyme inhibitors; ARBs, angiotensin receptor blockers; BMI, body mass index; CABG, coronary artery bypass graft; CCBs, calcium channel blockers; CCS, chronic coronary syndrome; CTO, chronic total occlusion; DAPT, dual antiplatelet therapy; DM, diabetes mellitus; eGFR, estimated glomerular filtration rate; FBG, fasting blood glucose; Hb, haemoglobin; HbA1c, haemoglobin A1c; HDL-C, high-density lipoprotein cholesterol; HsCRP, high-sensitivity C-reactive protein; IABP, intra-aortic balloon pump; ISR, in-stent restenosis; LDL-C, low-density lipoprotein cholesterol; LM, left main; LVEF, left ventricle ejection fraction; MI, myocardial infarction; NTEMI, non-ST segment elevated myocardial infarction; PCI, percutaneous coronary intervention; PLT, platelet; STEMI, ST segment elevated myocardial infarction; TC, total cholesterol; TG, triglycerides; UA, unstable angina; WBC, white blood cell.

^a^The data are presented as the means ± SDs, medians with interquartile ranges or *n* (%).

**Table 2:** Value of the CBR for predicting MACCE risk in different Cox proportional hazards models as a continuous variable.

Adjusted model	HR per SD increase	95% CI	*p*-Value
Crude model	1.12	1.06–1.19	<0.001
Model 1	1.12	1.06–1.18	<0.001
Model 2	1.10	1.04–1.17	0.001
Model 3	1.08	1.01–1.14	0.019

*Note:* Model 1 was adjusted for age and sex. Model 2 was adjusted for variables included in Model 1 and AMI, family history, previous MI, previous CABG, previous PCI, history of HTN, HL, DM, stroke, smoking status, LM disease, 3-vessel disease, CTO disease, SYNTAX score, profiles of stent implantation and IABP application. Model 3 was adjusted for variables included in Model 2 and LVEF, eGFR, WBC, PLT, Hb, HbA1c, FBG, TG, LDL-C, HDL-C, TC, uric acid and medication after discharge. CBR, hsCRP-to-BMI ratio.

Abbreviations: CI, confidence interval; HR, hazard ratio; MACCEs, major adverse cardiovascular and cerebrovascular events; SD, standard deviation.

**Table 3:** Value of the CBR for predicting MACCEs in different Cox proportional hazards models as a categorical variable.

Adjusted model	HR	95% CI	*p*-Value
Crude model			
Tertile 1	Reference	Reference	Reference
Tertile 2	1.16	0.99–1.35	0.07
Tertile 3	1.37	1.18–1.60	<0.001
Model 1			
Tertile 1	Reference	Reference	Reference
Tertile 2	1.15	0.98–1.34	0.085
Tertile 3	1.35	1.16–1.57	<0.001
Model 2			
Tertile 1	Reference	Reference	Reference
Tertile 2	1.13	0.97–1.33	0.126
Tertile 3	1.32	1.13–1.55	<0.001
Model 3			
Tertile 1	Reference	Reference	Reference
Tertile 2	1.14	0.96–1.34	0.131
Tertile 3	1.27	1.07–1.50	0.005

*Note:* Model 1 was adjusted for age and sex. Model 2 was adjusted for variables included in Model 1 and AMI, family history, previous MI, previous CABG, previous PCI, history of HTN, HL, DM, stroke, smoking status, LM disease, 3-vessel disease, CTO disease, SYNTAX score, profiles of stent implantation and IABP application. Model 3 was adjusted for variables included in Model 2 and LVEF, eGFR, WBC, PLT, Hb, HbA1c, FBG, TG, LDL-C, HDL-C, TC, uric acid and medication after discharge. CBR, hsCRP-to-BMI ratio.

Abbreviations: CI, confidence interval; HR, hazard ratio; MACCEs, major adverse cardiovascular and cerebrovascular events; SD, standard deviation.

## Data Availability

The data that support the findings of this study are available upon request from the corresponding author. The data are not publicly available due to privacy or ethical restrictions.

## Supporting Information

Supporting Information 1 Figure S1: Flowchart of the study population enrolment. Abbreviations: CAD, coronary artery disease; CBR, hsCRP-to-BMI ratio; DES, drug-eluting stent; eGFR, estimated glomerular filtration rate; hsCRP, high-sensitivity C-reactive protein. Figure S2. Kaplan‒Meier survival analysis of the secondary endpoint in the study population. (A) All-cause death; (B) MI; (C) stroke; (D) TVR. Abbreviations: CBR, hsCRP-to-BMI ratio; MI, myocardial infarction; TVR: target vessel revascularization. Figure S3. Restricted cubic spline of the association between the CBR and MI risk. The multivariable analysis model was adjusted for age, sex, AMI, family history, previous MI, previous CABG, previous PCI, history of HTN, HL, DM, stroke, smoking status, LM disease, 3-vessel disease, CTO disease, SYNTAX score, profiles of stent implantation, IABP application, LVEF, eGFR, WBC, PLT, Hb, HbA1c, FBG, TG, LDL-C, HDL-C, TC, uric acid and medication after discharge. Hazard ratios are indicated by solid lines, and 95% CIs are indicated by shaded areas. Figure S4. Sensitivity analyses of the association between the hsCRP-to-BMI ratio (CBR) and adverse outcomes across predefined subgroups. Description: this figure summarises robustness cheques referenced in the Results, including subgroup analyses (age, sex, diabetes, three-vessel disease and SYNTAX score strata) and alternative model specifications (with/without lipid covariates and renal function). Estimates are shown with 95% CIs. Methods and variable definitions are identical to those in the main text.

Supporting Information 2 Table S1: Value of the CBR for predicting MI in different Cox proportional hazards models as a continuous variable. Model 1 was adjusted for age and sex. Model 2 was adjusted for variables included in Model 1 and AMI, family history, previous MI, previous CABG, previous PCI, history of HTN, HL, DM, stroke, smoking status, LM disease, 3-vessel disease, CTO disease, SYNTAX score, profiles of stent implantation and IABP application. Model 3 was adjusted for variables included in Model 2 and LVEF, eGFR, WBC, PLT, Hb, HbA1c, FBG, TG, LDL-C, HDL-C, TC, uric acid and medication after discharge. Abbreviations: CBR, hsCRP-to-BMI ratio; CI, confidence interval; HR, hazard ratio; MACCEs, major adverse cardiovascular and cerebrovascular events; SD, standard deviation. Table S2. Value of the CBR for predicting MI in different Cox proportional hazards models as a categorical variable. Model 1 was adjusted for age and sex. Model 2 was adjusted for variables included in Model 1 and AMI, family history, previous MI, previous CABG, previous PCI, history of HTN, HL, DM, stroke, smoking status, LM disease, 3-vessel disease, CTO disease, SYNTAX score, profiles of stent implantation and IABP application. Model 3 was adjusted for variables included in Model 2 and LVEF, eGFR, WBC, PLT, Hb, HbA1c, FBG, TG, LDL-C, HDL-C, TC, uric acid and medication after discharge. Abbreviations: CBR, hsCRP-to-BMI ratio; CI, confidence interval; HR, hazard ratio; MACCEs, major adverse cardiovascular and cerebrovascular events; SD, standard deviation.
