# Supplementary material for: The Impact of the hsCRP/BMI Ratio on Cardiovascular Outcomes in CAD Patients: A Population-Based Study
Source: Mediators Inflamm. 2025 Nov 24;2025:6082331. doi: 10.1155/mi/6082331 (PMC12668850; doi:10.1155/mi/6082331)
Supplement: Supporting Information 1 — Figure S1: Flowchart of the study population enrolment. Abbreviations: CAD, coronary artery disease; CBR, hsCRP-to-BMI ratio; DES, drug-eluting stent; eGFR, estimated glomerular filtration rate; hsCRP, high-sensitivity C-reactive protein. Figure S2. Kaplan‒Meier survival analysis of the secondary endpoint in the study population. (A) All-cause death; (B) MI; (C) stroke; (D) TVR. Abbreviations: CBR, hsCRP-to-BMI ratio; MI, myocardial infarction; TVR: target vessel revascularization. Figure S3. Restricted cubic spline of the association between the CBR and MI risk. The multivariable analysis model was adjusted for age, sex, AMI, family history, previous MI, previous CABG, previous PCI, history of HTN, HL, DM, stroke, smoking status, LM disease, 3-vessel disease, CTO disease, SYNTAX score, profiles of stent implantation, IABP application, LVEF, eGFR, WBC, PLT, Hb, HbA1c, FBG, TG, LDL-C, HDL-C, TC, uric acid and medication after discharge. Hazard ratios are indicated by solid lines, and 95% CIs are indicated by shaded areas. Figure S4. Sensitivity analyses of the association between the hsCRP-to-BMI ratio (CBR) and adverse outcomes across predefined subgroups. Description: this figure summarises robustness cheques referenced in the Results, including subgroup analyses (age, sex, diabetes, three-vessel disease and SYNTAX score strata) and alternative model specifications (with/without lipid covariates and renal function). Estimates are shown with 95% CIs. Methods and variable definitions are identical to those in the main text. [file 6082331.f1.pptx]

## Slide 1
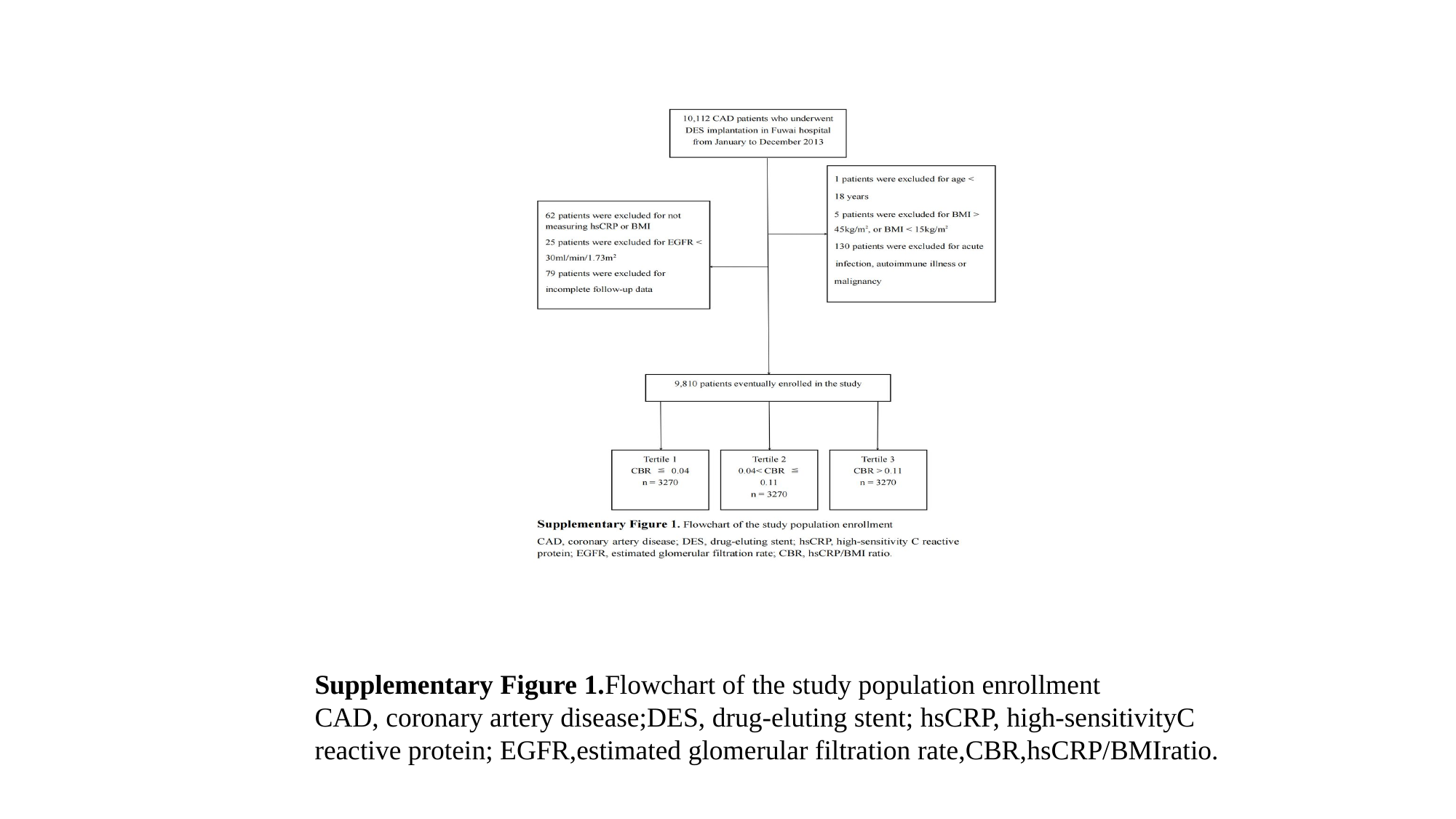

Supplementary Figure 1.Flowchart of the study population enrollment
CAD, coronary artery disease;DES, drug-eluting stent; hsCRP, high-sensitivityC reactive protein; EGFR,estimated glomerular filtration rate,CBR,hsCRP/BMIratio.

## Slide 2
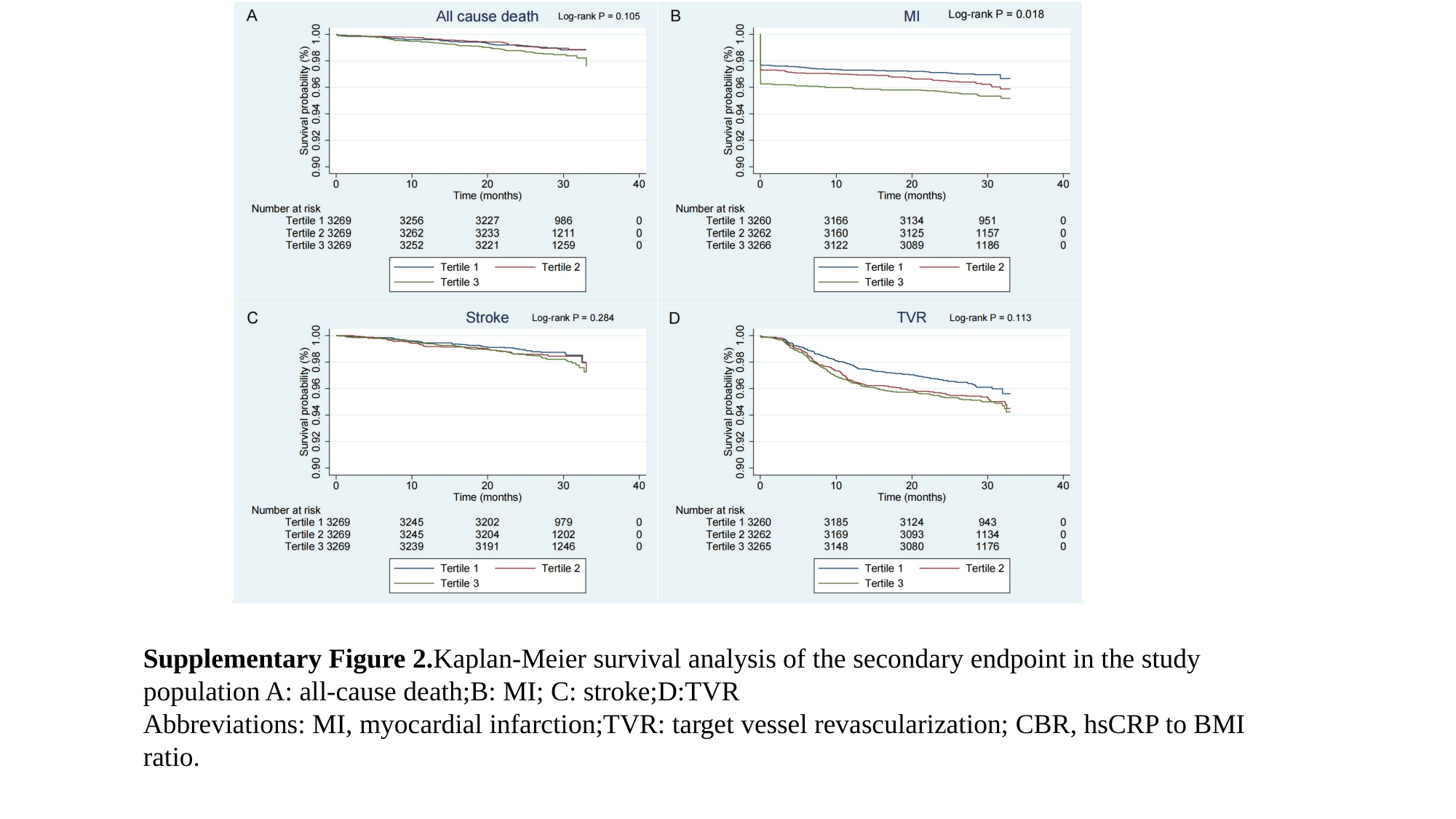

Supplementary Figure 2.Kaplan-Meier survival analysis of the secondary endpoint in the study population A: all-cause death;B: MI; C: stroke;D:TVR
Abbreviations: MI, myocardial infarction;TVR: target vessel revascularization; CBR, hsCRP to BMI ratio.

## Slide 3
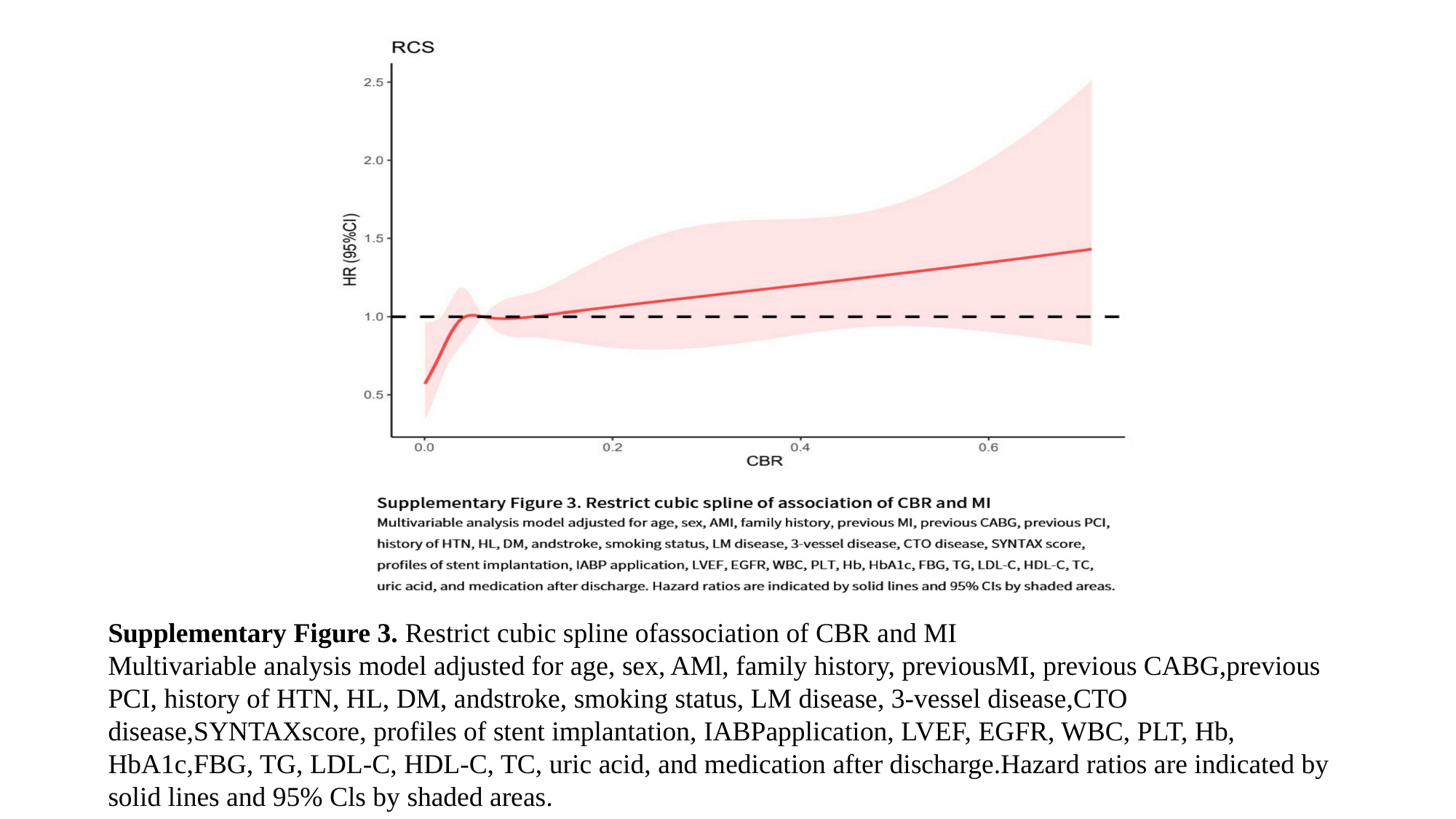

Supplementary Figure 3. Restrict cubic spline ofassociation of CBR and MI
Multivariable analysis model adjusted for age, sex, AMl, family history, previousMI, previous CABG,previous PCI, history of HTN, HL, DM, andstroke, smoking status, LM disease, 3-vessel disease,CTO disease,SYNTAXscore, profiles of stent implantation, IABPapplication, LVEF, EGFR, WBC, PLT, Hb, HbA1c,FBG, TG, LDL-C, HDL-C, TC, uric acid, and medication after discharge.Hazard ratios are indicated by solid lines and 95% Cls by shaded areas.

## Slide 4
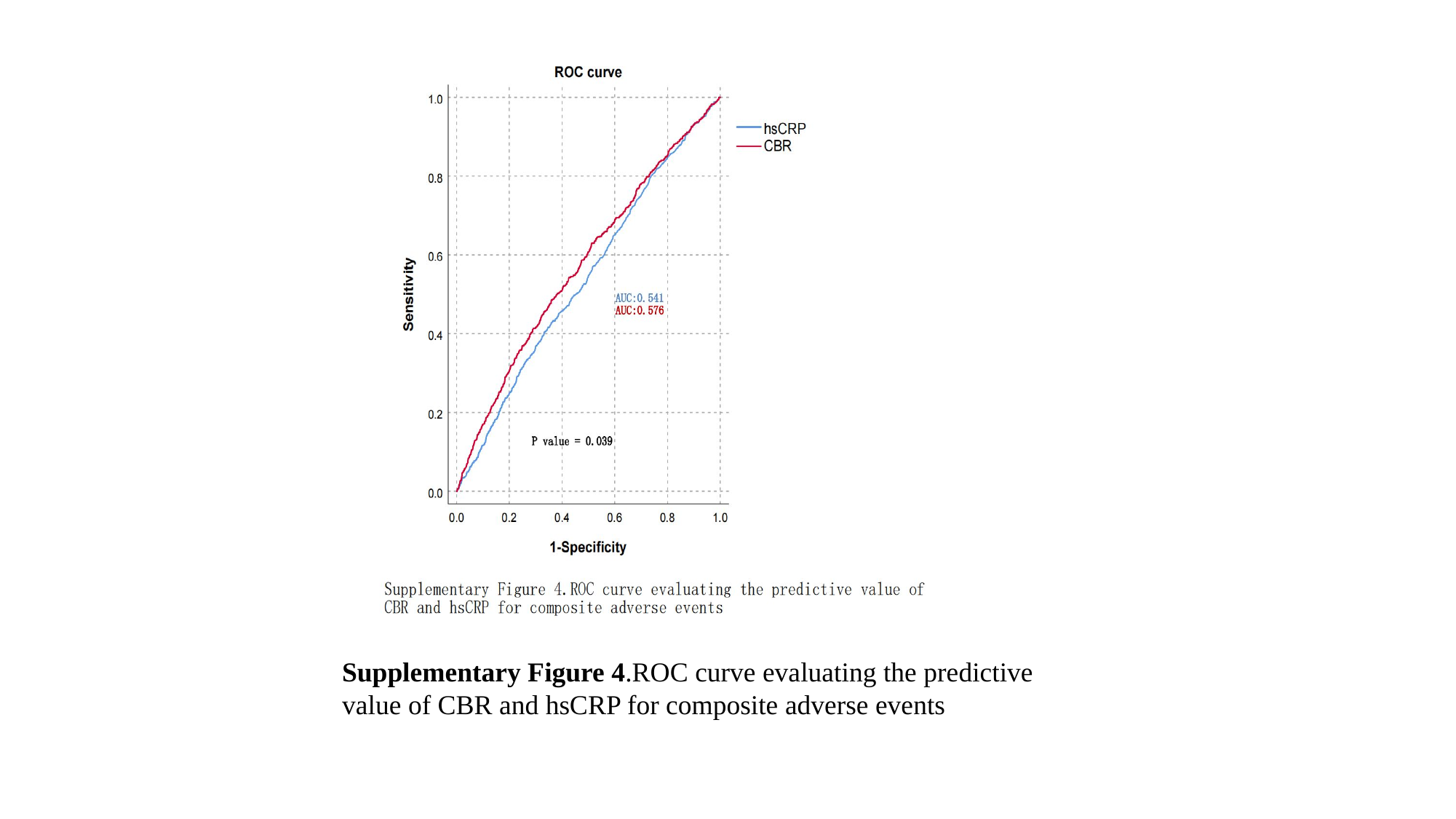

Supplementary Figure 4.ROC curve evaluating the predictive value of CBR and hsCRP for composite adverse events
