# Supplementary material for: The Impact of the hsCRP/BMI Ratio on Cardiovascular Outcomes in CAD Patients: A Population-Based Study
Source: Mediators Inflamm. 2025 Nov 24;2025:6082331. doi: 10.1155/mi/6082331 (PMC12668850; doi:10.1155/mi/6082331)
Supplement: Supporting Information 2 — Table S1: Value of the CBR for predicting MI in different Cox proportional hazards models as a continuous variable. Model 1 was adjusted for age and sex. Model 2 was adjusted for variables included in Model 1 and AMI, family history, previous MI, previous CABG, previous PCI, history of HTN, HL, DM, stroke, smoking status, LM disease, 3-vessel disease, CTO disease, SYNTAX score, profiles of stent implantation and IABP application. Model 3 was adjusted for variables included in Model 2 and LVEF, eGFR, WBC, PLT, Hb, HbA1c, FBG, TG, LDL-C, HDL-C, TC, uric acid and medication after discharge. Abbreviations: CBR, hsCRP-to-BMI ratio; CI, confidence interval; HR, hazard ratio; MACCEs, major adverse cardiovascular and cerebrovascular events; SD, standard deviation. Table S2. Value of the CBR for predicting MI in different Cox proportional hazards models as a categorical variable. Model 1 was adjusted for age and sex. Model 2 was adjusted for variables included in Model 1 and AMI, family history, previous MI, previous CABG, previous PCI, history of HTN, HL, DM, stroke, smoking status, LM disease, 3-vessel disease, CTO disease, SYNTAX score, profiles of stent implantation and IABP application. Model 3 was adjusted for variables included in Model 2 and LVEF, eGFR, WBC, PLT, Hb, HbA1c, FBG, TG, LDL-C, HDL-C, TC, uric acid and medication after discharge. Abbreviations: CBR, hsCRP-to-BMI ratio; CI, confidence interval; HR, hazard ratio; MACCEs, major adverse cardiovascular and cerebrovascular events; SD, standard deviation. [file 6082331.f2.docx]

| **Supplementary Table 1.** Predictive value of CBR for MI in different Cox proportional hazards models as a continuous variable | | | |
| --- | --- | --- | --- |
| **Adjusted model** | **HR per SD increase** | **95%CI** | **P value** |
| Crude model | 1.12 | 1.03-1.23 | 0.011 |
| Model 1 | 1.12 | 1.02-1.22 | 0.017 |
| Model 2 | 1.11 | 1.01-1.22 | 0.025 |
| Model 3 | 1.10 | 1.01-1.20 | 0.039 |

Model 1: adjusted for age and sex

Model 2: adjusted for variables included in Model 1 and AMI, family history, previous MI, previous CABG, previous PCI, history of HTN, HL, DM, and stroke, smoking status, LM disease, 3-vessel disease, CTO disease, SYNTAX score, profiles of stent implantation, and IABP application

Model 3: adjusted for variables included in Model 2 and LVEF, EGFR, WBC, PLT, Hb, HbA1c, FBG, TG, LDL-C, HDL-C, TC, uric acid, and medication after discharge

Abbreviations: CBR, hsCRP to BMI ratio; MACCE, major adverse cardiovascular and cerebrovascular events; HR, hazard ratio; SD, standard deviation; CI, confidential interval

| **Supplementary Table 2.** Predictive value of CBR for MI in different Cox proportional hazards models as a categorical variable | | | |
| --- | --- | --- | --- |
| **Adjusted model** | **HR** | **95%CI** | **P value** |
| Crude model |  |  |  |
| Tertile 1 | reference | reference | reference |
| Tertile 2 | 1.20 | 0.93-1.54 | 0.169 |
| Tertile 3 | 1.42 | 1.11-1.81 | 0.005 |
| Model 1 |  |  |  |
| Tertile 1 | reference | reference | reference |
| Tertile 2 | 1.18 | 0.91-1.52 | 0.208 |
| Tertile 3 | 1.39 | 1.09-1.77 | 0.009 |
| Model 2 |  |  |  |
| Tertile 1 | reference | reference | reference |
| Tertile 2 | 1.16 | 0.89-1.50 | 0.269 |
| Tertile 3 | 1.36 | 1.06-1.75 | 0.017 |
| Model 3 |  |  |  |
| Tertile 1 | reference | reference | reference |
| Tertile 2 | 1.14 | 0.88-1.48 | 0.311 |
| Tertile 3 | 1.32 | 1.03-1.71 | 0.027 |

Model 1: adjusted for age and sex

Model 2: adjusted for variables included in Model 1 and AMI, family history, previous MI, previous CABG, previous PCI, history of HTN, HL, DM, and stroke, smoking status, LM disease, 3-vessel disease, CTO disease, SYNTAX score, profiles of stent implantation, and IABP application

Model 3: adjusted for variables included in Model 2 and LVEF, EGFR, WBC, PLT, Hb, HbA1c, FBG, TG, LDL-C, HDL-C, TC, uric acid, and medication after discharge

Abbreviations: CBR, hsCRP to BMI ratio; MACCE, major adverse cardiovascular and cerebrovascular events; HR, hazard ratio; SD, standard deviation; CI, confidential interval
